# Isolation and phylogenomic characterization of two novel, dissimilar orthoreoviruses from Northern Alaskan Sea otters (*Enhydra lutris kenyoni*)

**DOI:** 10.1038/s41598-025-33400-0

**Published:** 2026-01-12

**Authors:** Justin P. Hawkins, Simon Anthony, Ole Nielsen, Kathy A. Burek Huntington, Natalie M. Rouse, Vsevolod L. Popov, Oliver Lung

**Affiliations:** 1 Canadian Food Inspection Agency, National Centre for Foreign Animal Disease, Winnipeg, MB Canada; 2 Department of Pathology, Microbiology, and Immunology, University of California Davis School of Veterinary Medicine, Davis, CA USA; 3 Department of Fisheries and Oceans Canada, 501 University Cr., Winnipeg, MB Canada; 4 Alaska Veterinary Pathology Services, 23834 The Clearing Drive, Eagle River, AK USA; 5 Institute for Human Infections and Immunity, The University of Texas Medical Branch, Galveston, TX USA; 6 Department of Biological Sciences, University of Manitoba, 50 Sifton Road, Winnipeg, MB R3T 2N2 Canada

**Keywords:** Northern sea otter (*E. lutris kenyoni*), Virus isolation, Genome sequencing, Phylogeny, Orthoreovirus, Marine mammal, Ecology, Ecology, Evolution, Genetics, Microbiology, Zoology

## Abstract

Sea otters are a keystone species with cultural significance to Indigenous populations. The increased mortality of Northern sea otters (*E. lutris kenyoni*) near Kachemak Bay, Alaska, led to a surveillance study to determine the cause of the increased deaths. Samples EL1562 and EL1411 were obtained from two dead-stranded sea otters. Whole genomes of two different orthoreoviruses with all ten segments were obtained. All EL1562 genome segments show the highest similarity to *Phocid orthoreovirus 1*, a new species isolated from harbor seals. However, the σ1 protein exhibits high divergence (34% amino acid identity) that warrants re-evaluation of the species demarcation criteria. Cell culture and microscopy are consistent with EL1562 being similar to *Phocid orthoreovirus 1*. Based on binomial naming conventions, we suggest the species name *Orthoreovirus marinarum*, which encompasses both EL1562 and PhRV1. Characterization of EL1411 sequences shows that it belongs to the species *Orthoreovirus mammalis*, which includes members from terrestrial mammals. This study represents the first orthoreovirus cultured and genome sequenced from sea otters, and is only the second report of a mammalian orthoreovirus in marine mammals. This study provides new insights into the genetic diversity of orthoreovirus in marine mammals and the evolutionary linkage between terrestrial and marine orthoreoviruses.

**Supplementary Information:**

The online version contains supplementary material available at 10.1038/s41598-025-33400-0.

## Introduction

The Northern sea otter subspecies (*E. lutris kenyoni*) is found along the Aleutian Islands, Southern Alaska, British Columbia, and Washington. They are the largest member of the *Mustelidae* with adult males reaching over five feet in length and weighing about 100 pounds. The population of northern sea otters was heavily reduced by commercial hunting that began in the 1700s, and when the first international conservation efforts were started in 1911, only an estimated few thousand remained. Sea otters were reintroduced to Southern Alaska between 1965 and 1969. Within Alaska, there are three stocks. The Southeast stock can be found in the coastal waters of Southeast Alaska. The Southcentral population spans from the west of Glacier Bay to the eastern edge of Cook Inlet, and the Southwest population stretches from the western edge of Cook Inlet along the Aleutian Islands. While the Southeast and south central populations are increasing sustainably, the Southwest stock is considered threatened under the Endangered Species Act (ESA) (https://www.adfg.alaska.gov/index.cfm?adfg=seaotter.main*).*

To aid in conservation efforts, a comprehensive investigation of sea otter mortalities in Alaska was undertaken from 2002 to 2012^1^. This study revealed that there was an unusually high number of subadult male sea otters dying from Streptococcal endocarditis, encephalitis and/or septicemia that were reported from Kachemak Bay^1^. Other threats detected include *Toxoplasma gondii* infection^2^, phocine distemper virus^3^, and poisoning from exposure to harmful algal bloom toxins^1^. A novel poxvirus has been isolated from skin lesions from individuals in both northern and southern (*Enhydra lutris neiris*) populations in Alaska and California, but it is not thought to have a significant negative impact on the health of sea otters at a population level^4^. Sea otters from Washington state are also known to have detectable IgG antibodies to several influenza A virus subtypes, indicating that sea otters are reservoir hosts of the virus^5^. SARS-CoV-2 infection has also been reported in the Wild European river otter (*Lutra lutra*)^6^. It is unknown whether sea otters are also susceptible to infection by SARS-CoV-2. To date, there are no reports in the scientific literature of any sea otters infected with reoviruses. However, there are reports of two species of reoviruses infecting and causing severe disease and death in a Steller sea lion (*Eumetopias jubatus*) from British Columbia, Canada^7^ and from the small outbreak of dying harbor seals (*Phoca vitulina*)^8^ in Washington state, USA.

The *Reoviridae* family is named for the acronym “reo” (respiratory enteric orphan) family of viruses. This family comprises viruses infecting humans, other mammals, birds, plants, and insects. Reoviruses do not have an envelope and possess segmented, double-stranded RNA genomes packaged within a double-shelled nucleocapsid of approximately 60–85 nm in diameter. While reoviruses are generally thought to cause asymptomatic or mild enteric and respiratory disease, some species can cause severe disease and death in humans^9^, other mammals and birds, including those of livestock importance^10^. Current research indicates that mammalian orthoreoviruses (MRVs) have an expanding host range and are increasingly recognized for their potential to cause severe disease, including in humans^11,12^.

Genetic diversity in viruses is driven through the accumulation of point mutations, reassortments, and recombination. These genetic changes produce RNA populations distinct from full-length genome segments and can influence viral population dynamics, persistence, and host immune responses. Viruses in the *Reoviridae* family, including rotavirus and mammalian orthoreoviruses, have been reported to package segments containing rearrangements or internal deletions, which can reduce the infectivity of the resulting viruses and alter their pathogenicity^13^. In viruses with segmented genomes, such as reoviruses, reassortment of genomic segments also contributes significantly to genetic drift^14^. Reassortment occurs when whole genomic segments of viruses are exchanged during co-infection of the same host. These events can impact the host range and pathogenicity, possibly leading to zoonotic transmission events^15^.

Disease surveillance and necropsies were conducted through the Alaska Marine Mammal Stranding Network by Alaska Veterinary Pathology Services and the Alaska SeaLife Center, with authorization from the United States Fish and Wildlife Service. From 2013 to 2015, both live and deceased animals were sampled, and live animals were also admitted for rehabilitation. Though some cases had pathology suggestive of a viral etiology, cultures and PCR tests for known viruses were negative, prompting further investigation by viral culture and high-throughput sequencing. Here, we describe the complete genome sequence and phylogeny of two novel but dissimilar viruses, both from this time period, representing a host range extension of mammalian orthoreovirus to mustelids. The pathogenicity of these viruses to sea otters is currently unknown. Both animals died during a distinct uptick (200%, Werner unpublished data) in sea otter deaths in this area (Homer, AK), and further investigation is warranted to determine the effect of this virus on sea otter mortality.

## Results

### Samples and pathologic findings

Samples in this study were from two individual northern sea otters. One was from feces of an abandoned female pup approximately 4 weeks of age (case EL1411) collected alive on June 14, 2014, who died 2 weeks later in a rehabilitation facility. In case EL1411, septicemia and acute suppurative bronchiolitis and bronchitis which was determined to be the terminal cause of death. The other sample was from a rectal swab of a female pup found dead in rigor on October 14, 2015 (case EL1562). Gross examination in case EL1562 found a normal body condition with a large amount of foam in the trachea, nose, and mouth, as well as widened interlobular septa and pulmonary edema. Histological examination confirmed pulmonary edema and mild splenic lymphoid hyperplasia. Initial PCR screening for paramyxovirus in both pups were negative.

### Cell culture of orthoreovirus isolate EL1562

**Fig. 1:**
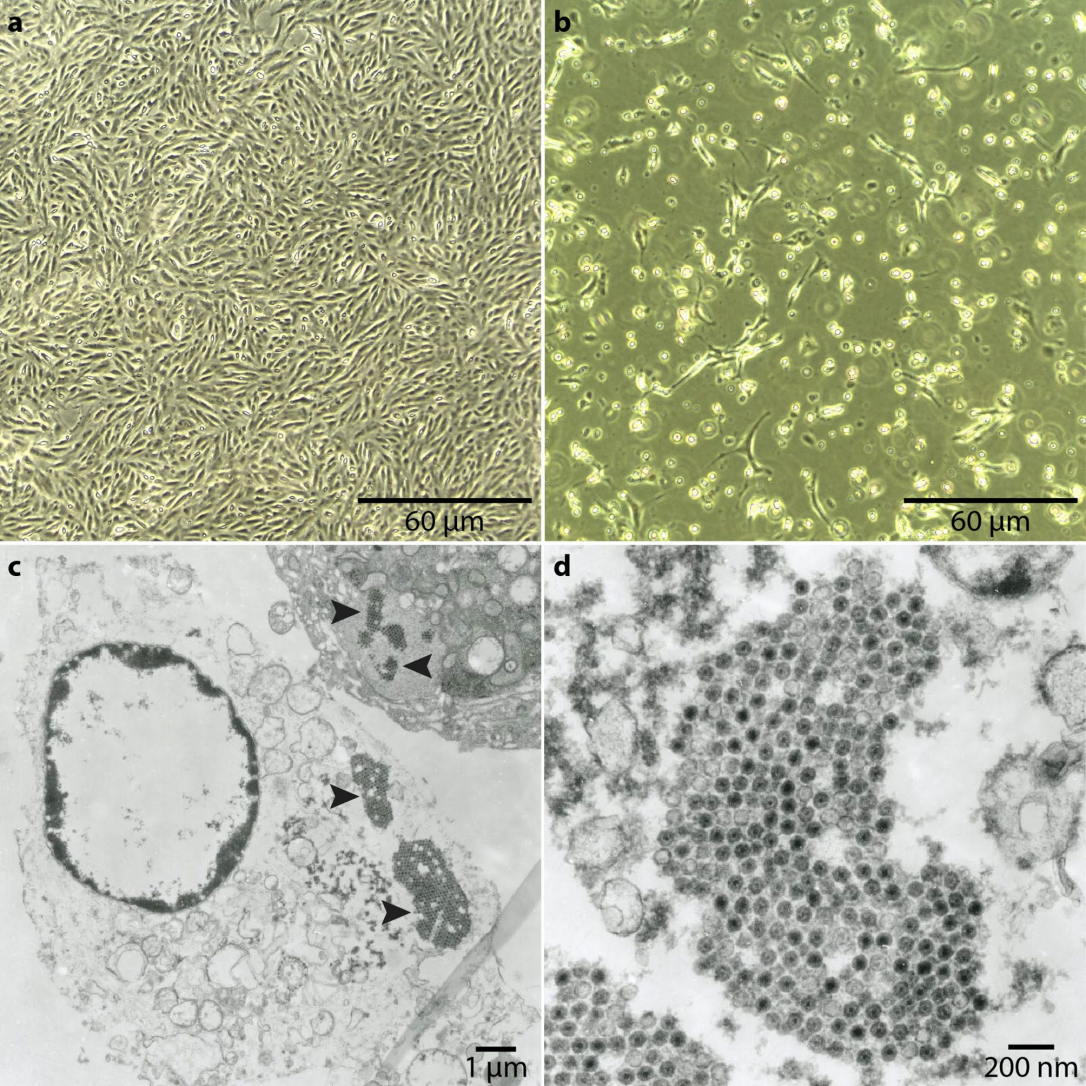
Viral culture of orthoreovirus from EL1562 using BWK cells. Cell culture (**a**, **b**) and transmission electron microscopy (**c**, **d**) of sea otter orthoreovirus strain EL1562 infecting BWK cells. (**a**) Uninfected BWK cells 5 DPI. (**b**) Passage 2 infected BWK cells 5 DPI showing extensive sub-complete monolayer destruction with no evidence of syncytial development. (**c**) Infected BWK and lysed BWK cells showing multiple clusters of orthoreovirus virions in the host cell cytosol. (**d**) Close-up of a cytosolic cluster of orthoreovirus virions ~60–65 nm in diameter.

Cell culture was used to propagate the virus from EL1562 to aid in the characterization and sequencing of the virus. Beluga whale kidney (BWK) and VeroDog.SLAMtag cells were inoculated with the rectal swab sample from EL1562. Approximately ten days post-infection (DPI), the flask containing inoculated BWK cells started to show signs of granularity, with some cells rounding up and detaching from the plastic (Fig. 1b). This progressed for a few days until most of the cells detached. Interestingly, some cells remained healthy in appearance and remained attached, but did not appear to be dividing. The VeroDog.SLAMtag flask inoculated with the rectal swab samples also showed similar signs of cytopathic effect (CPE) but appeared after 15 DPI. Significant CPE was observed upon passage in both cell lines in as little as four DPI. No signs of syncytia or cell fusion were noted in either cell line-infected flask. The cell control flasks showed no signs of CPE in either cell line, even after incubation and passage for six weeks (Fig. 1a). Overall, the results show successful infection and propagation in both cell lines.

### Transmission electron microscopy

In ultrathin sections of BWK cells infected with EL1562 rectal swab material on passage 2, most cells were lysed with destroyed organelles, but the plasma membrane and nucleus were preserved. Most of the cells were heavily infected and contained several clusters of reovirus virions formed from cytosolic virus factories (Fig. 1c). Virions were 60–65 nm in diameter and have a central dense core (Fig. 1d). These results taken with the observation of CPE are consistent with viruses within the family *Reoviridae.*

### Genome sequencing of isolates EL1562 and EL1411

**Fig. 2:**
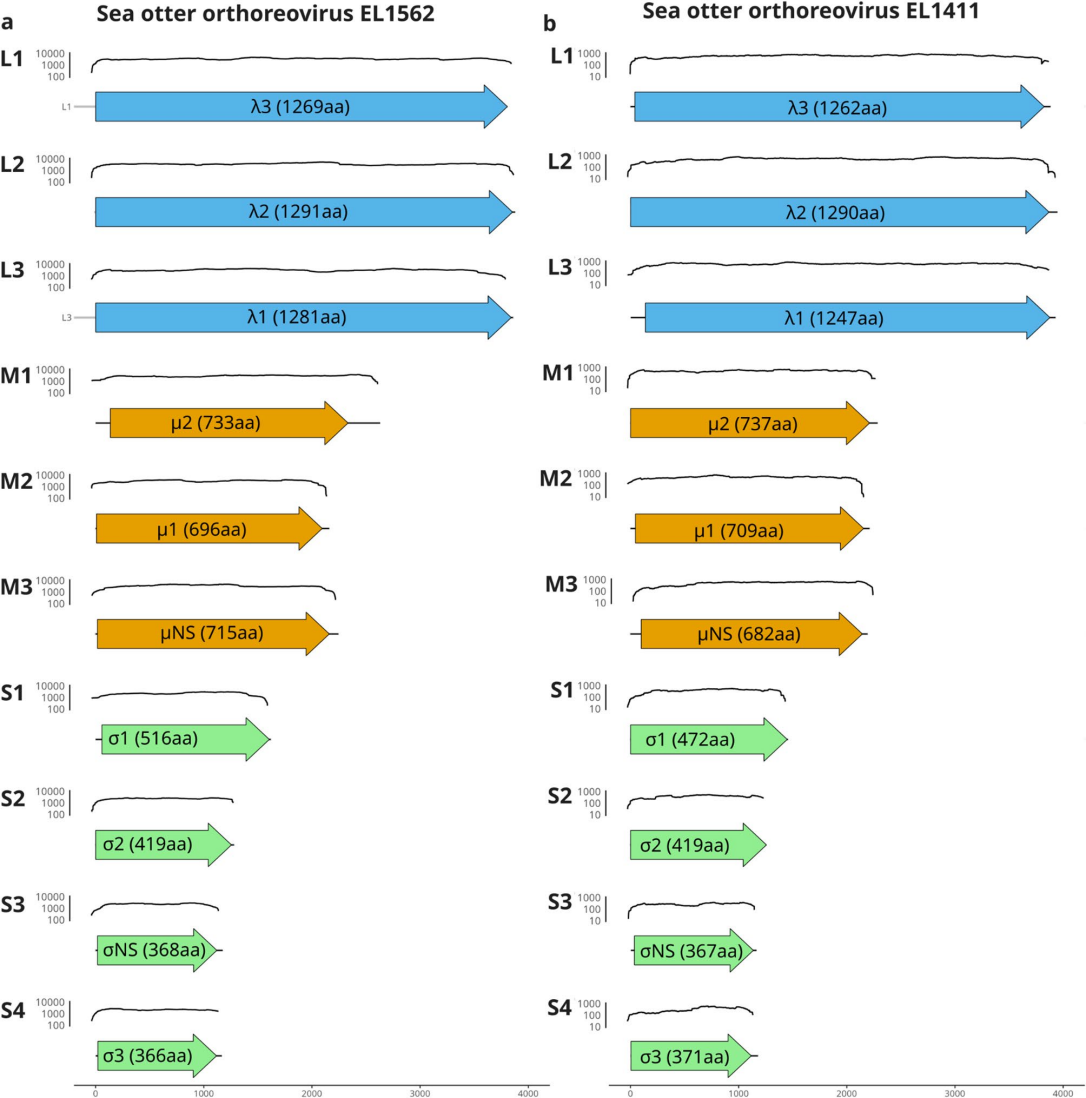
Genome segment configuration of the EL1562 and EL1411 orthoreovirus strains. Fold coverage and arrangement of sequenced genome segments from orthoreovirus strains EL1562 (**a**) and EL1411 (**b**). The line graph above each segment represents sequencing fold coverage in the area of the corresponding gene segment in log(10) scale. Scale bars at the bottom represent the total length of nucleotides present for each segment. Arrows indicate sequence within the predicted coding sequence present in each segment, while black lines indicate any potential sequence within 5’ and 3’ non-coding regions.

Whole genome sequencing of samples EL1562 and EL1411 was conducted to investigate the viral genomes present in each sample. The virus from sample EL1411 was directly sequenced from the swab sample, whereas isolate EL1562 was sequenced from the VeroDog.SLAMtag cell culture. Overall, 88,088 and 79,789 reads were used to assemble the genomes of EL1562 and EL1411, respectively. Ten contigs belonging to the genus *Orthoreovirus* were successfully assembled for each virus and investigated using BLASTX to determine viral species and segment function (Fig. 2a and b). The organization of the coding sequences for each segment was typical for *Orthoreovirus mammalis*, showing a single distinct coding sequence (CDS) per segment. Both genomes were also observed to lack a coding sequence for a fusion-associated transmembrane protein. This is a known feature of MRVs and *Orthoreovirus piscis* and is consistent with the absence of observed cell fusion events during cell culture (Fig. 1b).

All genome segments belonging to orthoreovirus EL1562 showed the highest nucleotide identity to a putative new species of orthoreovirus (*phocid orthoreovirus 1*, PhRV1) isolated from a deceased stranded harbor seal in 2007, with identities ranging from 72 to 93% (Table 1)^8^. However, the CDS for σ1 (outer fiber protein) showed high divergence from PhRV1 σ1, with only 46.3% pairwise nucleotide and 34.2% pairwise amino acid (aa) identity between the two sequences aligned using MAFFT. While the structure of the gene segments largely resembled those of PhRV1, the coding sequences for µ2 (core NTPase) and σ2 (core clamp protein) from EL1562 had truncated reading frames in comparison. When the two µ2 genes are aligned, a (c.2T > G) single nucleotide polymorphism (SNP) in EL1562 would abolish the start codon used in PhRV1. This difference moves the putative EL1562 µ2 start codon 60 nucleotides downstream of the original PhRV1. In addition, an EL1562 µ2 SNP (c.2257G > T) compared to PhRV1 caused a premature stop codon 462 nucleotides upstream of the corresponding PhRV1 stop codon. The result is a putative truncated µ2 protein of 733 aa compared to the 894 aa of PhRV1. In EL1562 σ2, a (c.103G > A) SNP compared to PhRV1 creates a start codon (ATG). A stop codon was also identified 9 nucleotides upstream from this ATG, and again at the start of the segment. These changes would result in a putative σ2 protein truncated at the N-terminal portion of the protein that is 419 aa compared to the 452 aa observed in PhRV1.

**Table 1:** Sea otter orthoreovirus strain EL1562 CDS characterization.

CDS	Putative function	Size (bp)	Top BLAST Hit	Accession Number	Nucleotide identity	Amino acid identity
λ1	RNA binding, NTPase, helicase	3843	PhRV1	MN820529.1	93.1%	99.3%
λ2	Guanyltransferase, methyltransferase	3873	PhRV1	MN820530.1	86.3%	96.2%
λ3	RNA-dependent RNA polymerase	3807	PhRV1	MN820531.1	89.9%	98.4%
µ1	Cell penetration, transcriptase activation	2088	PhRV1	MN820532.1	72.6%	83.2%
µ2*	Binds RNA NTPase	2199	PhRV1	MN820533.1	74.2%	95.1%
µNS	Non-structural, RNA binding, phosphoprotein	2145	PhRV1	MN820534.1	86.5%	94.5%
σ1	Cell attachment, type-specific antigen	1548	PhRV1	MN820535.1	46.3%	34.2%
σ2*	dsRNA binding	1257	PhRV1	MN820536.1	93.6%	99.5%
σ3	dsRNA binding, IFN response modulation	1098	PhRV1	MN820537.1	88.4%	97.0%
σNS	ssRNA binding, inclusion body formation	1104	PhRV1	MN820538.1	91.5%	95.9%

Most genome segments from strain EL1411 had sequences from mammalian orthoreovirus 1 as the top BLAST hit based on e-value and bit score (Table 2). All CDSs shared high pairwise nucleotide (91.0–98.9%) and aa (96.2–99.9%) identities using MAFFT to their closest identified mammalian orthoreovirus coding sequence, as determined by BLAST analysis. Six of the ten segments exhibit the highest identity to mammalian orthoreovirus serotype 1 strain SI-MRV06, which was previously isolated from fruit bats in Slovenia^16^. Another two genes (σ2 and σNS) also showed the highest identity to mammalian orthoreovirus 1, but to sequences obtained initially from bats in Italy^17^. While most segments had higher identity to MRV serotype 1, the sequences for λ1 and µ2 (core shell protein and core NTPase) had top blast hits to MRV serotype 2 isolated from humans. While the top BLAST hits are listed here (Tables 1 and 2), it is important to note that other top BLAST hits for genome segments in EL1411 were often within 1% identity and were limited to sequences publicly available on NCBI. No insertions or truncations were observed when comparing EL1411 CDSs to their best BLAST hits.

**Table 2:** Sea otter orthoreovirus strain EL1411 CDS characterization.

CDS	Putative function	Size (bp)	Top BLAST Hit	Accession Number	Nucleotide identity	Amino acid identity
λ1	RNA binding, NTPase, helicase	3828	MRV2	LC476917.1	97.7%	99.6%
λ2	Guanyltransferase, methyltransferase	3865	MRV1	MG457119.1	98.5%	99.1%
λ3	RNA-dependent RNA polymerase	3786	MRV1	MG457118.1	98.1%	99.4%
µ1	Cell penetration, transcriptase activation	2156	MRV1	MG457122.1	98.8%	99.9%
µ2	Binds RNA NTPase	2207	MRV2	AY428874.1	91.0%	96.2%
µNS	Non-structural, RNA binding, phosphoprotein	2166	MRV1	MG457123.1	98.5%	99.4%
σ1	Cell attachment, type-specific antigen	1449	MRV1	MG457124.1	98.6%	98.3%
σ2	dsRNA binding	1277	MRV1	KT900702.1	95.0%	97.8%
σ3	dsRNA binding, IFN response modulation	1113	MRV1	MG457127.1	98.9%	99.7%
σNS	ssRNA binding, inclusion body formation	1136	MRV1	KT900703.1	98.8%	99.7%

### Phylogenetic and genetic analyses

**Fig. 3:**
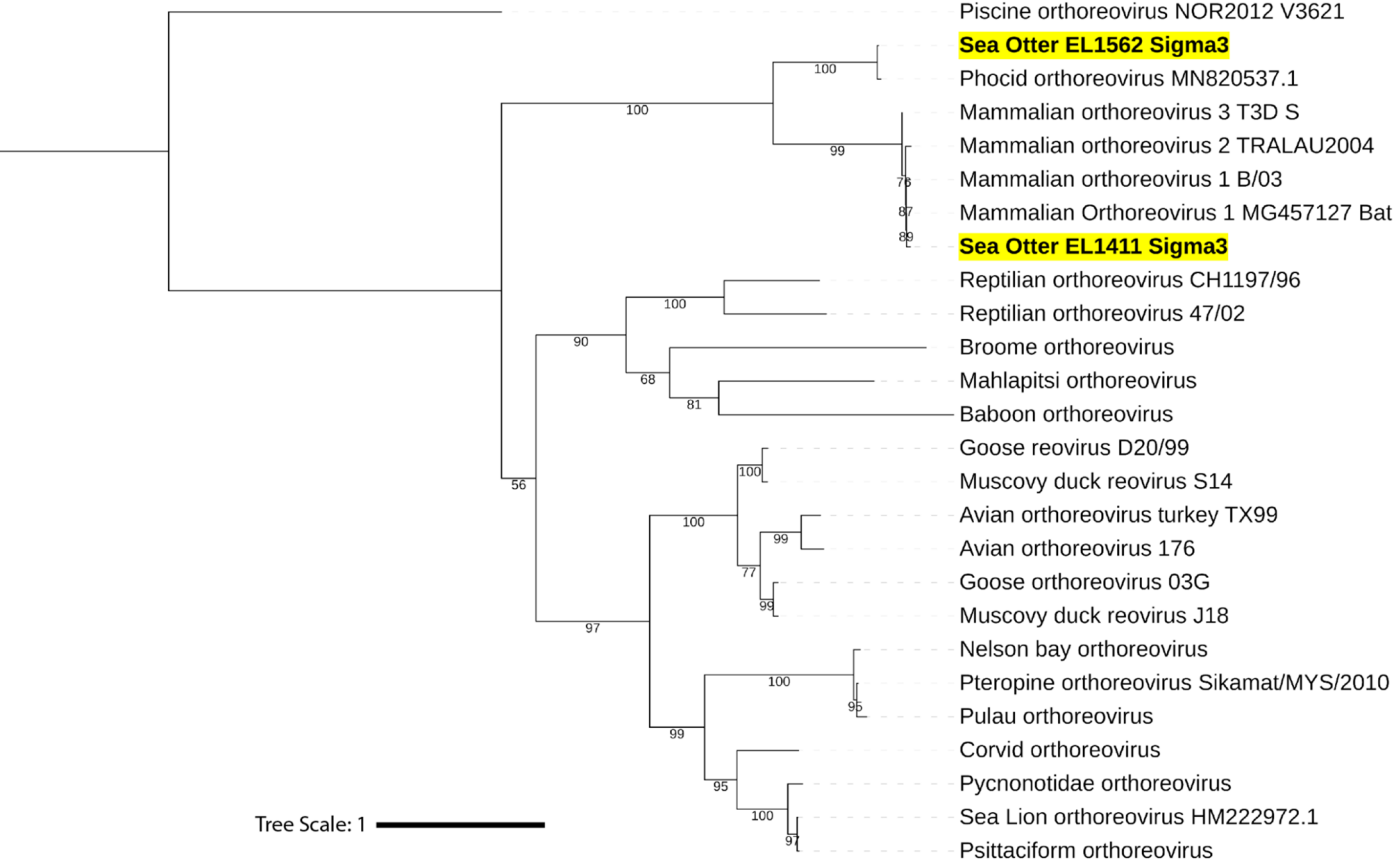
Phylogenetic typing of σ3 amino acid sequences from sea otter isolates. Maximum likelihood phylogenetic tree of representative strains from representative orthoreovirus species. The tree was constructed based on the amino acid sequence alignment of the outer clamp protein for each indicated strain. Novel sequences are indicated in bold with yellow highlights. Phylogenetic analysis was performed using IQ-TREE and visualized with iTOL, using piscine orthoreovirus NOR2012 V3621 as the outgroup. The scale bar represents the estimated average number of aa substitutions per site.

**Fig. 4:**
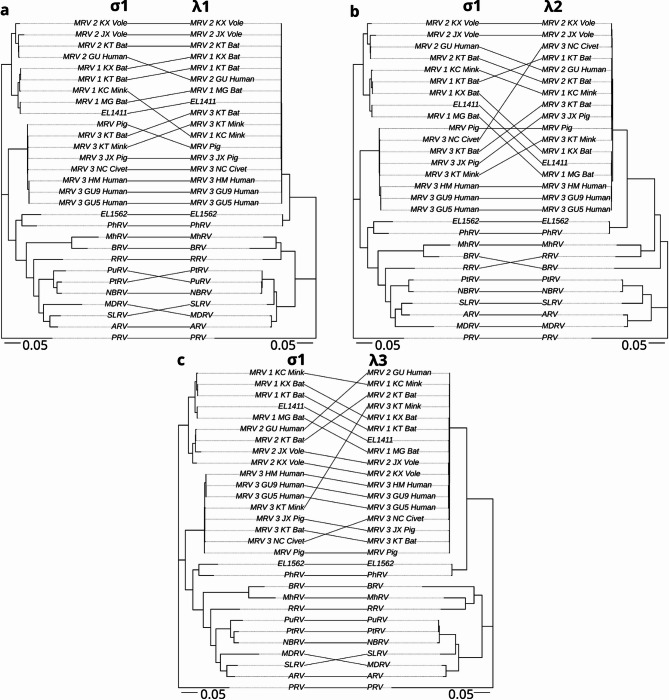
Phylogenetic tanglegrams of sea otter orthoreovirus λ proteins compared to σ1 amino acid sequences. Tanglegrams of representative strains with whole genomes from representative orthoreovirus species of λ 1,2, and 3 (**a**–**c**). Trees were constructed based on the amino acid sequence alignment of the indicated protein. Phylogenetic analysis was performed using IQTree and tanglegrams were visualized with R, using piscine orthoreovirus isolate CGA280-05 as the outgroup. The scale bar represents the estimated average number of aa substitutions per site. Accession numbers of sequences used in analysis can be found in Table S11. Abbreviations: MRV: Mammalian orthoreovirus, PRV: Piscine orthoreovirus, PtRV: Pteropine orthoreovirus, PuRV: Pulau orthoreovirus, BRV: Baboon orthoreovirus, MhRV: Mahlapitsi orthoreovirus, SLRV: Sea lion orthoreovirus, NBRV: Nelson Bay orthoreovirus, RRV: Reptilian orthoreovirus, ARV: Avian orthoreovirus, PhRV: Phocid orthoreovirus.

**Fig. 5:**
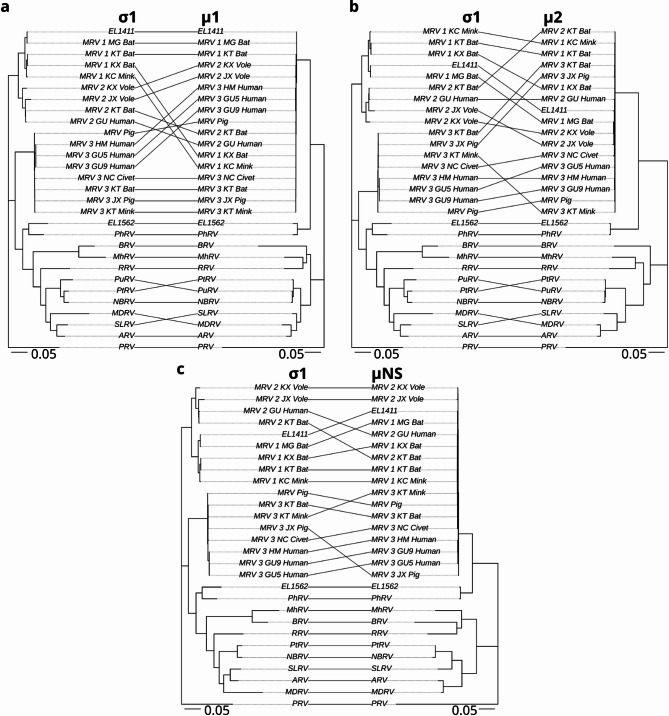
Phylogenetic tanglegrams of sea otter orthoreovirus μ proteins compared to σ1 amino acid sequences. Tanglegrams of representative strains with whole genomes from representative orthoreovirus species of μ 1,2, and NS (**a**–**c**). Trees were constructed based on the amino acid sequence alignment of the indicated protein. Phylogenetic analysis was performed using IQTree and tanglegrams were visualized with R, using piscine orthoreovirus isolate CGA280-05 as the outgroup. The scale bar represents the estimated average number of aa substitutions per site. Accession numbers of sequences used in analysis can be found in Table S11. Abbreviations: MRV: Mammalian orthoreovirus, PRV: Piscine orthoreovirus, PtRV: Pteropine orthoreovirus, PuRV: Pulau orthoreovirus, BRV: Baboon orthoreovirus, MhRV: Mahlapitsi orthoreovirus, SLRV: Sea lion orthoreovirus, NBRV: Nelson Bay orthoreovirus, RRV: Reptilian orthoreovirus, ARV: Avian orthoreovirus, PhRV: Phocid orthoreovirus.

**Fig. 6:**
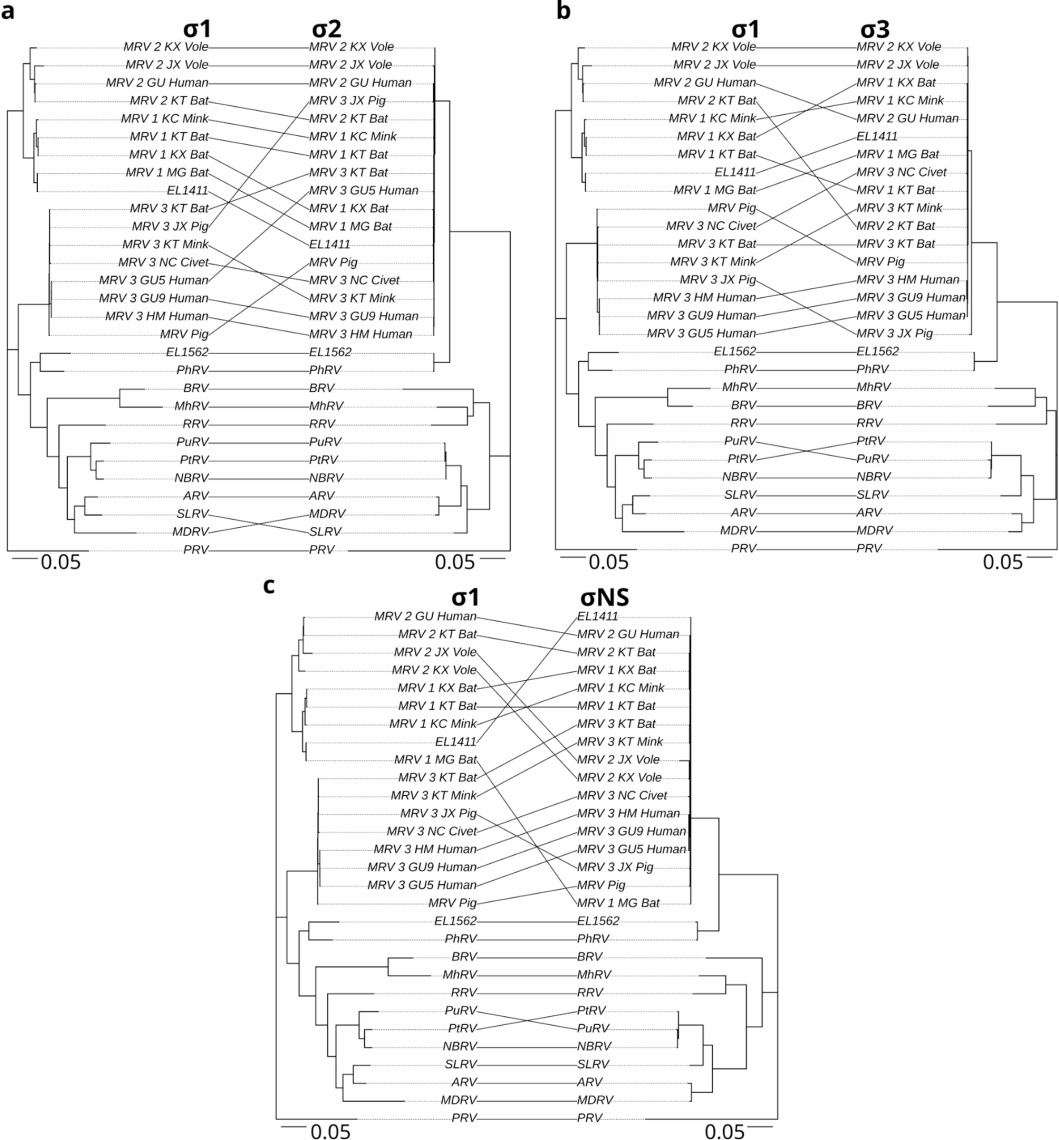
Phylogenetic tanglegrams of sea otter orthoreovirus σ proteins compared to σ1 amino acid sequences. Tanglegrams of representative strains with whole genomes from representative orthoreovirus species of σ 2, 3, and NS (**a**–**c**). Trees were constructed based on the amino acid sequence alignment of the indicated protein. Phylogenetic analysis was performed using IQTree and tanglegrams were visualized with R, using piscine orthoreovirus isolate CGA280-05 as the outgroup. The scale bar represents the estimated average number of aa substitutions per site. Accession numbers of sequences used in analysis can be found in Table S11. Abbreviations: MRV: Mammalian orthoreovirus, PRV: Piscine orthoreovirus, PtRV: Pteropine orthoreovirus, PuRV: Pulau orthoreovirus, BRV: Baboon orthoreovirus, MhRV: Mahlapitsi orthoreovirus, SLRV: Sea lion orthoreovirus, NBRV: Nelson Bay orthoreovirus, RRV: Reptilian orthoreovirus, ARV: Avian orthoreovirus, PhRV: Phocid orthoreovirus.

Initial phylogenetic analysis of viruses EL1562 and EL1411 was performed using the outer clamp protein (σ3) aa sequences obtained from International Committee on the Taxonomy of Viruses (ICTV) (https://ictv.global/ accessed 2025-01-21) for orthoreovirus species typing (Fig. 3). EL1411 clustered to MRV 1 strain B/03 obtained from short-nosed Indian fruit bats (*Cynopterus sphinx*)^18^. EL1562 formed a well-supported sister clade to the MRV sequences and clustered along with the σ3 sequence from PhRV1. These findings, along with the observed best BLAST hits, led to the designation of the EL1411 virus as MRV 1 and EL1562 as belonging to the same species as PhRV1 (putatively named *Orthoreovirus marinarum*). Further analysis of each individual genome segment was carried out using additional MRV sequences to classify each protein sequence (Figs. 4, 5 and 6). EL1411 sequences consistently formed tight clusters with MRV sequences. EL1411 σ1 (outer fiber protein) aa sequence also clustered with MRV serotype 1 sequences, further corroborating EL1411 as MRV 1. The aa identity of EL1411 virus σ1 was also observed to have the highest identity to the MRV 1 σ1 sequences tested compared to other MRV sequences (~ 79% aa identity to MRV1) (Table S7). However, in the nine other proteins, no distinction between MRV serotypes was readily visible as aa identity among MRV sequences was observed to be similar between serotypes 1, 2, and 3 (Tables S1–S10). Segments of EL1411 were assessed for potential reassortment using tanglegrams comparing phylogenies of each segment to σ1 (Figs. 4, 5 and 6). No evidence of reassortment was seen in each segment for the tested strains as it was determined EL1411 clustered closest with that of MRV1 isolated from bats (MRV1_MG_Bat).

Sequences from the EL1562 virus consistently formed the same clade with PhRV1 as a sister clade to the MRV sequences for all ten genome segments (Figs. 4, 5 and 6, Fig. S1a–S10a). This held true even in the case of σ1, where significant divergence between the two sequences was observed (34.2% aa sequence identity). All ten proteins of the EL1562 virus and PhRV1 consistently had the highest pairwise aa identity to each other out of all sequences (Fig. S1b–S10b).

The EL1562 virus σ1 protein was further investigated due to its low pairwise aa identity to other known orthoreovirus σ1 proteins. InterPro^19^ (https://www.ebi.ac.uk/interpro/) and Phyre2.2^20^ (https://www.sbg.bio.ic.ac.uk/phyre2/) were used to determine if any conserved domains or predicted structures were present in the protein. InterPro revealed a conserved domain consistent with the C-terminal of σ1 reovirus proteins (325–509 aa) and a virus attachment globular domain (368–510 aa). In addition, Phyre2.2 predicted conserved domains of the orthoreovirus σ1 protein from Mammalian orthoreovirus strains T1L and T3D throughout the entirety of the protein with low identity (32% and 18%, respectively). These results were consistent with the EL1562 virus having σ1 functionality despite the high sequence divergence. No other conserved domains were detected in the protein using the described methods.

## Discussion

This work describes the detection and genomic characterization of two different orthoreoviruses from sea otters from Homer, Alaska, in 2014 and 2015. The whole-genome sequence of both viruses was obtained and revealed that these viruses belong to, or are closely related to, the mammalian orthoreoviruses. While both avian orthoreovirus and mammalian-like orthoreovirus have been isolated from Steller sea lion and harbor seals, this study represents the first reported culturing and whole-genome sequencing of orthoreovirus from sea otters^7,8^. In addition, this would be only the second reported isolation of *Orthoreovirus mammalis* from marine mammals^8^.

Reoviruses are known to cause infections of varying degrees of severity in the respiratory and gastrointestinal tracts, as well as the central nervous system^21^. Specifically, mammalian orthoreovirus is known to infect many terrestrial hosts and cause several different types of infections. Clinical signs include gastrointestinal, respiratory, and nervous system infections that could lead to pneumonia, meningitis, encephalitis, and enteritis^21–23^. It has also been suggested that MRV may act synergistically with other infections^24^. While the genomes of the two orthoreovirus strains are the only viruses detected in the fecal swabs from these sea otters, the pathogenicity of the orthoreoviruses and whether they directly contributed to the mortality event is unknown. The EL1562 virus displayed mostly similar genome segments, cell culture, and viral morphology traits as the previously isolated PhRV1 from harbor seals. Interestingly, PhRV1 was isolated from a number of organs, including the brain and lungs of many dead harbor seals. However, no consistent signalment, gross pathology, histopathology, or ancillary diagnostic findings were identified or associated with PhRV1 infection^8^. Due to the similarities of the viruses, this could imply that the EL1562 orthoreovirus may also infect multiple organs in sea otters and contribute to mortality if associated with significant pathology. However, like in the case for PhRV1, further study would be required to prove the role of EL1562 orthoreovirus in mortality.

Orthoreoviruses can be categorized into fusogenic and non-fusogenic orthoreoviruses, with non-fusogenic cultures being a trait so far observed to be unique to *Orthoreovirus picsis* and *Orthoreovirus mammalis*^25,26^. The observation of non-fusogenic cultures while culturing isolate EL1562 is consistent with phylogenetic and pairwise identity observations indicating that EL1562 and PhRV1 are closely related to mammalian orthoreovirus. Mammalian orthoreoviruses are known to infect and cause disease in a wide array of hosts^27^. Not only was the EL1562 virus isolated from a sea otter, but it could also be propagated and demonstrated infection of BWK cells (derived from beluga) and VeroDog.SLAMtag cells (derived from an African green monkey). This implies that the EL1562 virus has an extensive host range spanning both terrestrial and marine mammals, further supporting the hypothesis that the EL1562 virus is closely linked to mammalian orthoreovirus.

*Phocid orthoreovirus 1* is a novel orthoreovirus species closely related to but distinct from mammalian orthoreovirus. The current criteria set out by ICTV suggests that > 85% aa identity between the core proteins (λ1–3, µ2, and σ2), and > 55% between outer capsid proteins (µ1, σ1, and σ3) indicates the sequences belong to the same species, whereas < 65% and < 35%, respectively, indicate belonging to separate species^28^. The orthoreovirus from isolate EL1562 exhibits high pairwise identity with PhRV1, displaying 83.2–99.5% aa identity for each protein, except σ1, which displayed only 34.2% pairwise identity to PhRV1 σ1 (Table 1). In comparison, the best pairwise aa identity of the core and outer capsid proteins from the EL1562 virus when compared to mammalian orthoreoviruses ranged from 43.7–77.5% and 28.2–59.6%, respectively (Table S1-S10). Based on these criteria and phylogenetic clustering with PhRV,1 with the exception of σ1 (Figs. 4, 5 and 6, S1-S10), EL1562 appears to belong to the same orthoreovirus species as PhRV1 and is distinct from MRV. As this species does not have a formal binomial name we suggest *Orthoreovirus marinarum* to encompass these orthoreoviruses. However, ICTV’s species demarcation criteria may need to be re-evaluated due to the low identity between the two σ1 proteins. In contrast, EL1411 displayed high identity to mammalian orthoreovirus segments and clustered with MRV1 based on phylogenetic analysis of σ1.

The viral attachment protein σ1 from isolate EL1562 showed unexpectedly high aa sequence divergence compared to σ1 from other orthoreoviruses (34.2% pairwise identity to PhRV1, 22.5–27.8% to MRV orthoreoviruses). Its low pairwise identity to known orthoreovirus σ1 proteins may indicate this segment is a reassortant from an undiscovered novel orthoreovirus species. In orthoreoviruses, σ1 is an outer fiber protein that plays a key role in cell attachment. The involvement in cell attachment implies a role in determining viral host range^29^. The origin of the highly divergent S1 gene segment is unknown but may be due to a reassortment with an uncharacterized orthoreovirus. Further investigation of the σ1 protein revealed homology to domains present in σ1 from other orthoreoviruses, including a C-terminal globular domain. This domain is essential for reoviruses and adenoviruses as it modulates the binding of the viral attachment protein to cell surface receptors such as junction adhesion molecules. The presence of the C-terminal globular domain suggests that while there is low sequence identity to other orthoreovirus σ1 proteins, the core functionality of the protein is likely intact. Since σ1 mediates host cell attachment, high sequence divergence could indicate that the EL1562 virus has a different host range than other known orthoreoviruses. This hypothesis is consistent with the EL1562 virus being isolated from a sea otter, a mustelid, though it shares a high identity with PhRV1 isolated initially from harbor seals, a phocid. Further investigation is needed to determine the origin of the S1 segment and the host range of the EL1562 virus.

Orthoreoviruses are genetically diverse, infecting a large variety of hosts worldwide while also displaying a propensity for host switching^28,30,31^. These characteristics, combined with its large number of genome segments, facilitate opportunities for segment reassortment and recombination that can lead to the generation of variants that can cross-species barriers. Mammalian orthoreovirus also displays a lack of species barrier, with a single virus able to infect an array of hosts^11^. EL1411 contains eight segments with top BLAST hits to MRV serotype 1, and two with top hits to MRV serotype 2, suggesting these two segments (L3 and M1) may have been acquired through reassortment. Recombination and reassortment between animal and human orthoreoviruses raises concerns about the potential for zoonotic transmission. While events of zoonotic transmission of orthoreovirus from bats to humans have been reported, there is no evidence of viral persistence in humans from these cases^23,32,33^. However, it is important to consider that mammalian orthoreoviruses from other hosts may adapt to efficiently infect humans, especially with evidence of recombination between human and animal orthoreoviruses.

Sea otters are considered sentinel species of marine ecosystems, serving as early warning signs of environmental change or emerging diseases. While it is unclear if the viruses in this study are the root cause of increased mortality events of sea otters in Alaska, detecting these viruses indicates a novel health challenge to marine mammals in the environment and local communities that harvest sea otters for subsistence. Sea otters are also preyed upon by sea lions, killer whales, and sharks, thus serving as a potential entry vector for these viruses into other marine mammals. In addition to the previously mentioned isolations of orthoreovirus from Steller sea lions and harbor seals, orthoreovirus has putatively been detected from a metagenomic screen of a fecal sample from a New Zealand sea lion (Unpublished, accession number PV335598.1). Whole-genome sequencing helped reveal significant changes in the σ1 protein of the EL1562 virus, which may be a direct result of host adaptation of the virus for other marine mammals or recombination from yet undiscovered marine orthoreoviruses. Further investigation and resources are necessary to study the impact of orthoreovirus on marine mammals and determine the extent of the spread of the virus through various marine mammal species.

## Materials and methods

### Animals and pathological examinations

Case # 1 EL1562 was a female sea otter pup found dead, floating and in rigor in the Homer boat harbor, Homer, AK (59.6054 N, 151.4255 W) on October 14, 2015. The carcasses were collected under USFWS permit no. FWS/AIA/DMA/LOA-837,414. Gross necropsy was performed using standard protocols^34^. Rectal swabs were collected in 2 mL cryovials with viral transport media (Remel) and frozen at − 20 °C (~ 1 week) and then at − 80 °C (~ 5 months) before being shipped on dry ice for viral culture. Fixed tissues were processed at Histological Consulting Services (Everson, WA) and stained with hematoxylin and eosin for interpretation by a veterinary pathologist.

Case # 2 EL1411 was a female (~ 4 weeks) sea otter pup found live and abandoned near Homer, AK (59.59968 N, 151.41693 W) on June 14, 2014, and transported to the Alaska SeaLife Center (Seward, AK) for rehabilitation under USFWS permit no. FWS/AIA/DMA/LOA-837,414. Samples were collected on admit, including feces in a 15 mL conical tube and frozen at − 80 °C. The pup died 2 weeks later and was necropsied using standard protocols^34^. Fixed tissues were processed at Histological Consulting Services (Everson, WA) and stained with hematoxylin and eosin for interpretation by a veterinary pathologist. The fecal sample taken on admission to rehabilitation was shipped directly to the NGS lab on dry ice for sequencing.

### Virus isolation

Routine virus isolation of sample EL1562 was done using two cell lines that had previously proven useful in isolating viruses from marine mammals. The continuous cell line VeroDog.SLAMtag was originally developed from the Vero cell line to isolate and study morbilliviruses^35^, including a novel cetacean morbillivirus from a Fraser’s dolphin (*Lagenodelphis hosei*) from Hawaii^36^, a novel alphavirus from a harbor porpoise (*Phocoena phocoena*) from Alaska^37^, and a novel orthoreovirus from a limited outbreak of mortality in harbor seals (*Phoca vitulina*) from Washington state^8^. A finite primary cell line derived from beluga (*Delphinapterus leucas*) kidney has also been used successfully to isolate Senecavirus isolates from a dead harbor porpoise and a beluga whale, also from Alaska^38^. Briefly, the rectal swab EL1562 was thawed quickly and 0.5 ml was directly inoculated into a 70% confluent 25 cm^2^ tissue culture flask of each cell line, and a sample of Hank’s Balanced Salts Solution containing antibiotics (penicillin 200 International Units/ml, streptomycin 200 mg/ml, gentamicin 50 mg/ml, and 0.25 mg/ml Fungizone) constituted the cell control. The inoculum was allowed to adsorb for 1 h at 37 °C, then decanted, and a mixture of 5.0 ml of cell culture medium (DMEM/F-12), fetal calf serum (10% for BWK and 4% for VeroDog.SLAMtag) and antibiotics were added. Flasks were incubated at 37 ℃ and observed daily for CPE. Flasks were subcultured weekly for 6 weeks before being discarded as negative. When CPE could be successfully diluted and passaged, presumptive virus isolates were harvested at passage two and frozen at − 80 °C for further study. Virus isolation was not attempted for EL1411.

### Transmission electron microscopy

A 70% confluent culture of BWK cells in a 75 cm^2^ flask was infected with a 1:100 dilution of a flask inoculated with the rectal swab sample of EL1562, showing extensive CPE. After incubation and subsequent development of CPE, the infected cells were fixed in 15.0 ml of Karnovsky’s fixative (2% formaldehyde plus 2% glutaraldehyde in 0.1 M cacodylate buffer, pH 7.4) for 2 h at room temperature. The cells were harvested in 0.1 M Cacodylate buffer, pelleted, and post-fixed in 1% OsO_4_ in 0.1 M cacodylate buffer pH 7.4. The sample was stained *en bloc* with 2% aqueous uranyl acetate (20 min at 60 °C), dehydrated through a graded ethanol series, infiltrated and embedded in epoxy resin PolyBed 812 (Polysciences, Inc., Warrington, PA, United States). Ultrathin Sects. (60–90 nm) were cut on a Leica EM UC7 ultramicrotome and stained with lead citrate prior to examination in a JEOL JEM 1400 electron microscope at 80 kV.

### Oxford nanopore sequencing for isolate EL1562

To extract nucleic acid from cultured cells infected with sample EL1562 at the Canadian Food Inspection Agency National Centre for Foreign Animal Disease (Winnipeg, Manitoba, Canada), 1.0 ml of the sample was subjected to two rounds of freeze-thaw at − 20 °C. The sample was centrifuged at 13,000 RPM for 1 min, and the supernatant was removed for viral RNA extraction. RNA was extracted using the Zymo Quick-DNA/RNA miniprep plus kit (ZymoBIOMICS) following the protocol provided by the manufacturer with the optional on-column DNase I treatment. To improve cDNA synthesis from dsRNA, samples were first denatured at 98 °C for 10 min and then immediately snap-cooled at 4 °C for a minimum of one minute. Denatured RNA was then used for first-strand cDNA synthesis using the Superscript IV First-Strand Synthesis System (ThermoFisher) following the manufacturer’s recommendation, with modification of the reverse transcription time at 55 °C to 90 min. Second-strand synthesis was then carried out using the NEBNext Ultra II Non-Directional RNA Second Strand Synthesis Module (NEB) following the manufacturer’s recommendations. The resulting dsDNA was then cleaned using AMPure beads (Beckman Coulter) at a 1.8X concentration and eluted in ddH_2_O for sequencing.

Nanopore library preparation was performed using the Native Barcoding Kit 96 V14 (Oxford Nanopore) according to the recommended protocol and third-party reagents. The final library was loaded onto an R10.4.1 flow cell and sequenced on a Nanopore Mk1C using MinKNOW v23.07.12 for 24 h. Following sequencing, base-calling was performed using Dorado v0.8.3 with the super-accurate base-calling model.

### Illumina sequencing and contig assembly for EL1411

To sequence sample EL1411, RNA was extracted from the fecal sample using QIAamp Viral RNA Mini Kit (Qiagen) according to the manufacturer’s protocol. Libraries were prepared using the Twist Total Nucleic Acids Library Preparation EF Kit 2.0 (Twist Bioscience), following the manufacturer’s custom protocol for viral detection and characterization. This workflow accommodates all nucleic acid types (ssRNA, dsRNA, ssDNA, dsDNA) and includes a denaturation step followed by random priming, reverse transcription, and second-strand synthesis. Resulting double-stranded cDNA and DNA were subjected to enzymatic fragmentation, end repair, A-tailing, ligation with Twist Universal Adapters, and PCR amplification using Twist Unique Dual Index (UDI) primers. Libraries were sequenced on an Illumina NovaSeq X platform using 150 bp paired-end reads, generating 43.5 M reads. *De novo* assembly was performed using SPAdes v4.2.0^39^, and the assembled contigs were investigated using BLASTX searches using the nr database. Initial putative reading frames for all 10 segments of the MRV EL1411 strain were predicted using GeneMarkS^40^.

### Bioinformatic analysis

The resulting FastQ reads for EL1562 were processed using an in-house built pipeline and run using Nextflow v24.04.4. In brief, sequencing adaptors were trimmed using Porechop ABI v0.5.0^41^, and Fastplong v0.2.1^42^ was used to filter reads based on a Q score of at least 7 and a length of 50 base pairs. Reads were then classified using Kraken2 v2.1.3^43^ using the NCBI core_nt index compiled September 04, 2024 (downloaded from https://benlangmead.github.io/aws-indexes/k2), followed by filtering for reads that were classified as viral or unclassified using KrakenTools v1.2 to remove host and background reads. Filtered reads were then assembled using Flye v2.9.4, Canu v2.2, and MetaMDBG v1.0^44–46^. Flye was run using the “--meta” flag for metagenomic assemblies, and Canu was used with settings also suggested for metagenomic assemblies. Finally, assembled contigs were queried against the BLAST non-redundant (nr) protein sequences database (downloaded December 28, 2023) using DIAMOND v2.1.10 to identify viral contigs^47^.

The Geneious overlap-based assembler v2024.0.3 was used to complete coding sequences and obtain sequences for 5′/3′ UTRs for both EL1562 and EL1411, where necessary. This was done iteratively to extend each contig until a complete coding sequence was predicted by Geneious ORF Finder v2024.0.3, or total fold coverage dropped below 10x as predicted by Minimap2 v2.24^48^. Final contigs were confirmed using Minimap2 by mapping reads against the final product to ensure a minimum of 10-fold coverage and that no errors were made in the iterative process. This same process was used to complete contigs from EL1411 that had incomplete CDS at the 5′ and 3′ ends of the segment. Coding sequences from isolates EL1562 and EL1411 were then examined using BLASTX (https://blast.ncbi.nlm.nih.gov/*)* with the nr database to identify the closest related protein sequence and segment for each contig (Tables 1 and 2).

### Species identification

To determine the species of *Orthoreovirus* identified, the putative aa sequence for the outer clamp protein (σ3) was compared to those from other *Orthoreovirus* species reported on ICTV (https://ictv.global/*).* The sequences of phocid orthoreovirus 1 strain PhRV1^8^ and Mammalian orthoreovirus 1 strain SI-MRV06^16^ were also included in this analysis as they were the closest related strains to the EL1562 and EL1411 strains. σ3 typing sequences (*n* = 23) were downloaded from ICTV and aligned with query sequences using ClustalO (v1.2.3)^49^. Initial species determination was carried out based on the criteria that sequences within the same species should share greater than 65% amino acid identity, and those with less would be considered a different species, as suggested by the ICTV. Phylogenetic analysis for identifying orthoreovirus species was conducted using IQ-TREE v2.3.4^50^ on the ClustalO alignment files, using automatic model determination^51,52^ and 1000x bootstrapping, and visualized with iTOL (https://itol.embl.de/)^53^.

### Phylogenetic analysis

After strains were identified as mammalian and phocid orthoreovirus, each segment from strains EL1562 and EL1411 was compared against a more comprehensive panel of mammalian orthoreovirus amino acid sequences (*n* = 31 for λ1–3, µ2, µNS, and σNS; *n* = 32 for µ1, σ1–3) that were previously used in the identification and characterization of phocid orthoreovirus 1^8^. All sequences were grouped by segment and then aligned using MAFFT v7.49^54^. The resulting files were analyzed with IQ-TREE v2.3.4^50^ to generate maximum-likelihood phylogenies using automatic model determination^51,52^ and 1000x ultrafast bootstrapping. Phylogenetic trees were rooted to piscine orthoreovirus and visualized using iTOL (https://itol.embl.de/)^53^. Alignment heatmaps were generated using the Sequence Demarcation Tool v1.3 with MAFFT alignment of aa sequences^55^. Tanglegrams comparing phylogenetic arrangement of strains from each segment was constructed using R. The accession numbers of each sequence used in the tanglegrams can be found in Table S11.

## Supplementary Information

Below is the link to the electronic supplementary material.

Supplementary Material 1

Supplementary Material 2

## Data Availability

Genomes for PhRV1/Enhydra lutris/USA/2015/EL1562 and MRV1/Enhydra lutris/USA/2014/EL1411 have been deposited under BioProject ID PRJNA1256812 and BioSample ID numbers SAMN48198720 and SAMN48198757. Genome assembly and Sequence read archive accession numbers are as follows: EL1562 (PV591111-PV591120 and SRR33371489); EL1411 (PV818064-PV818073 and SRR34063982).
