# Supplementary material for: Isolation and phylogenomic characterization of two novel, dissimilar orthoreoviruses from Northern Alaskan Sea otters (Enhydra lutris kenyoni)
Source: Sci Rep. 2026 Jan 12;16:3511. doi: 10.1038/s41598-025-33400-0 (PMC12847709; doi:10.1038/s41598-025-33400-0)
Supplement: Supplementary file 1 — Supplementary Material 1 [file 41598_2025_33400_MOESM1_ESM.docx]

**Isolation and Phylogenomic Characterization of Two Novel, Dissimilar Orthoreoviruses from Northern Alaskan Sea Otters (*Enhydra lutris kenyoni*)**

**Justin P. Hawkins^1^, Simon Anthony^2^, Ole Nielsen^3^, Kathy A. Burek Huntington^4^, Natalie M. Rouse^4^, Vsevolod L. Popov^5^, Oliver Lung^1, 6^**

**^1^ Canadian Food Inspection Agency, National Centre for Foreign Animal Disease, Winnipeg, MB, Canada.**

**^2^ Department of Pathology, Microbiology, and Immunology, University of California Davis School of Veterinary Medicine, Davis, California, United States of America**

**^3^ Department of Fisheries and Oceans Canada, 501 University Cr. Winnipeg, Manitoba, Canada**

**^4^Alaska Veterinary Pathology, 23834 The Clearing Drive, Eagle River, Alaska, USA**

**^5^ Institute for Human Infections and Immunity, The University of Texas Medical Branch, Galveston, TX, USA**

**^6^ Department of Biological Sciences, 50 Sifton Road, University of Manitoba, Winnipeg, Manitoba, R3T 2N2 Canada**

**^*^Corresponding author:  Oliver Lung, Canadian Food Inspection Agency, National Centre for Foreign Animal Disease, Winnipeg, MB, Canada. e-mail:** [**Oliver.Lung@inspection.gc.ca**](mailto:Oliver.Lung@inspection.gc.ca)

**Fig S1.** λ**1 protein phylogenetic analysis**

**
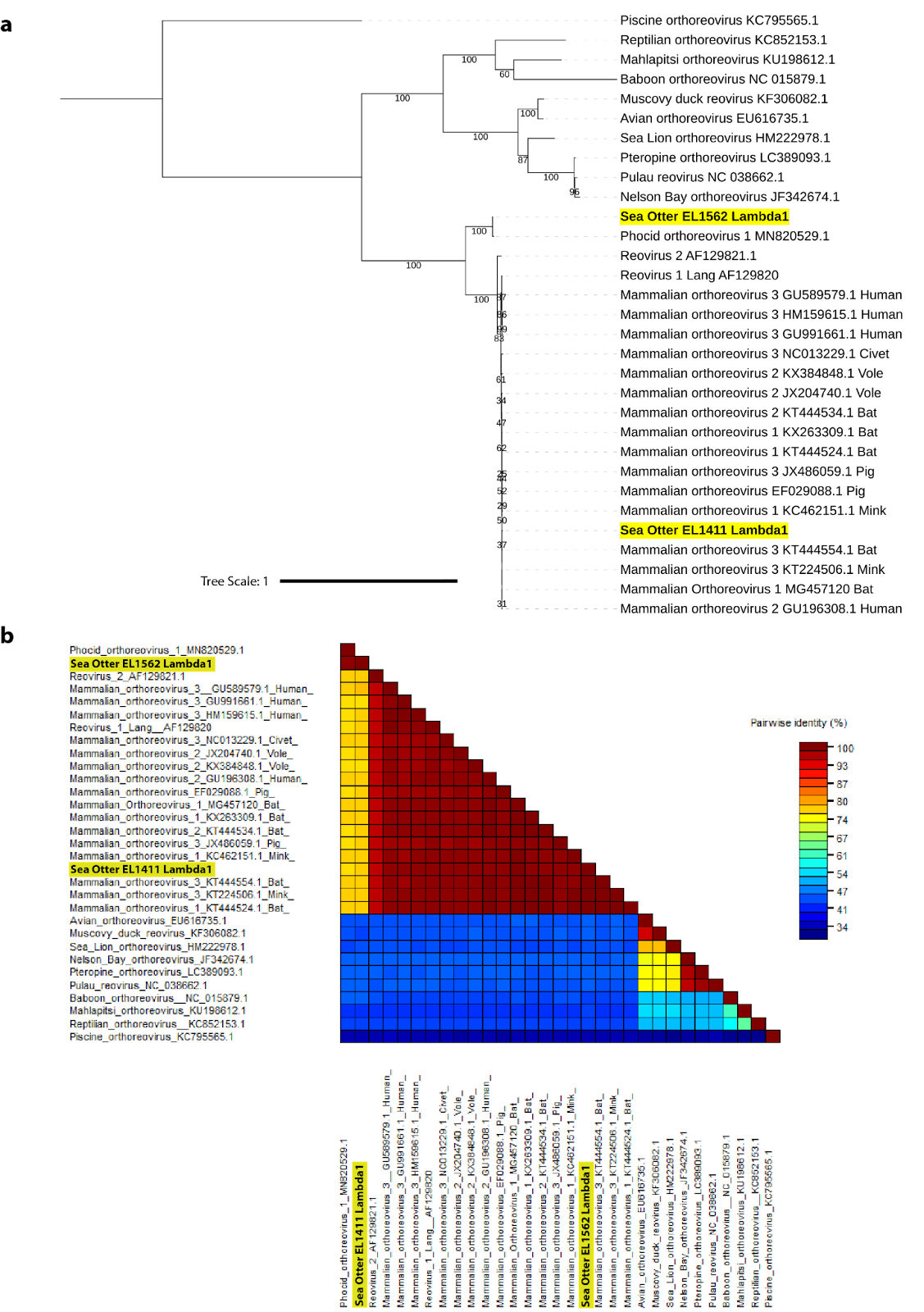
**

Phylogenetic and amino acid identity analysis of sea otter orthoreovirus λ1 amino acid sequences. Maximum likelihood phylogenetic tree of representative strains from representative orthoreovirus species. The tree was constructed based on the amino acid identity of the core shell protein for each indicated strain. Novel sequences are indicated in bold with yellow highlights. Phylogenetic analysis was performed with IQTree and visualized with iTOL using Piscine orthoreovirus isolate CGA280-05 as the outgroup. The scale bar represents the estimated average number of aa substitutions per site.

**Fig S2.** λ**2 protein phylogenetic analysis**

**
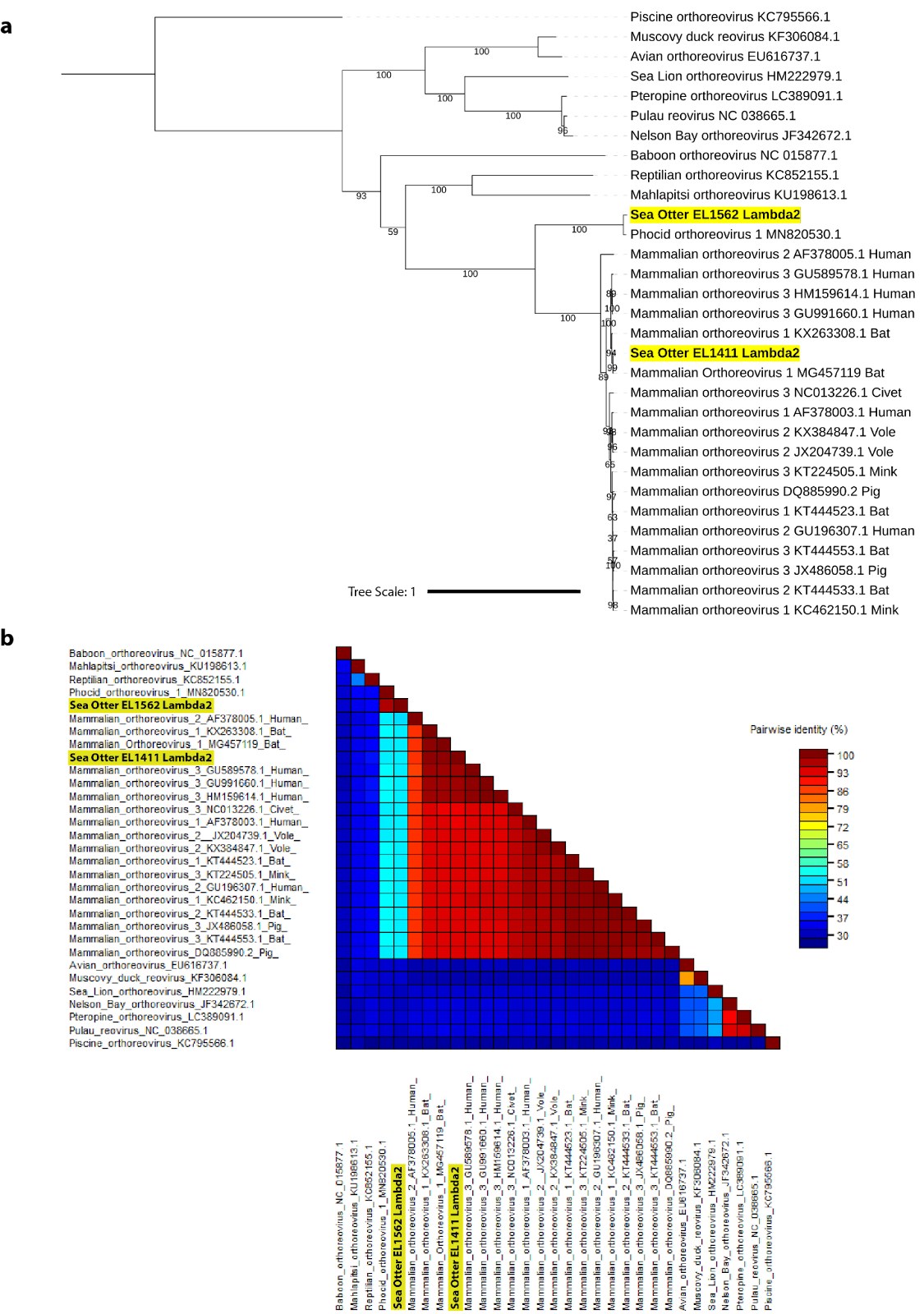
**

Phylogenetic and amino acid identity analysis of sea otter orthoreovirus λ2 amino acid sequences. Maximum likelihood phylogenetic tree of representative strains from representative orthoreovirus species. The tree was constructed based on the amino acid identity of the core turret protein for each indicated strain. Novel sequences are indicated in bold with yellow highlights. Phylogenetic analysis was performed with IQTree and visualized with iTOL using Piscine orthoreovirus isolate CGA280-05 as the outgroup. The scale bar represents the estimated average number of aa substitutions per site.

**Fig S3.** λ**3 protein phylogenetic analysis**

**
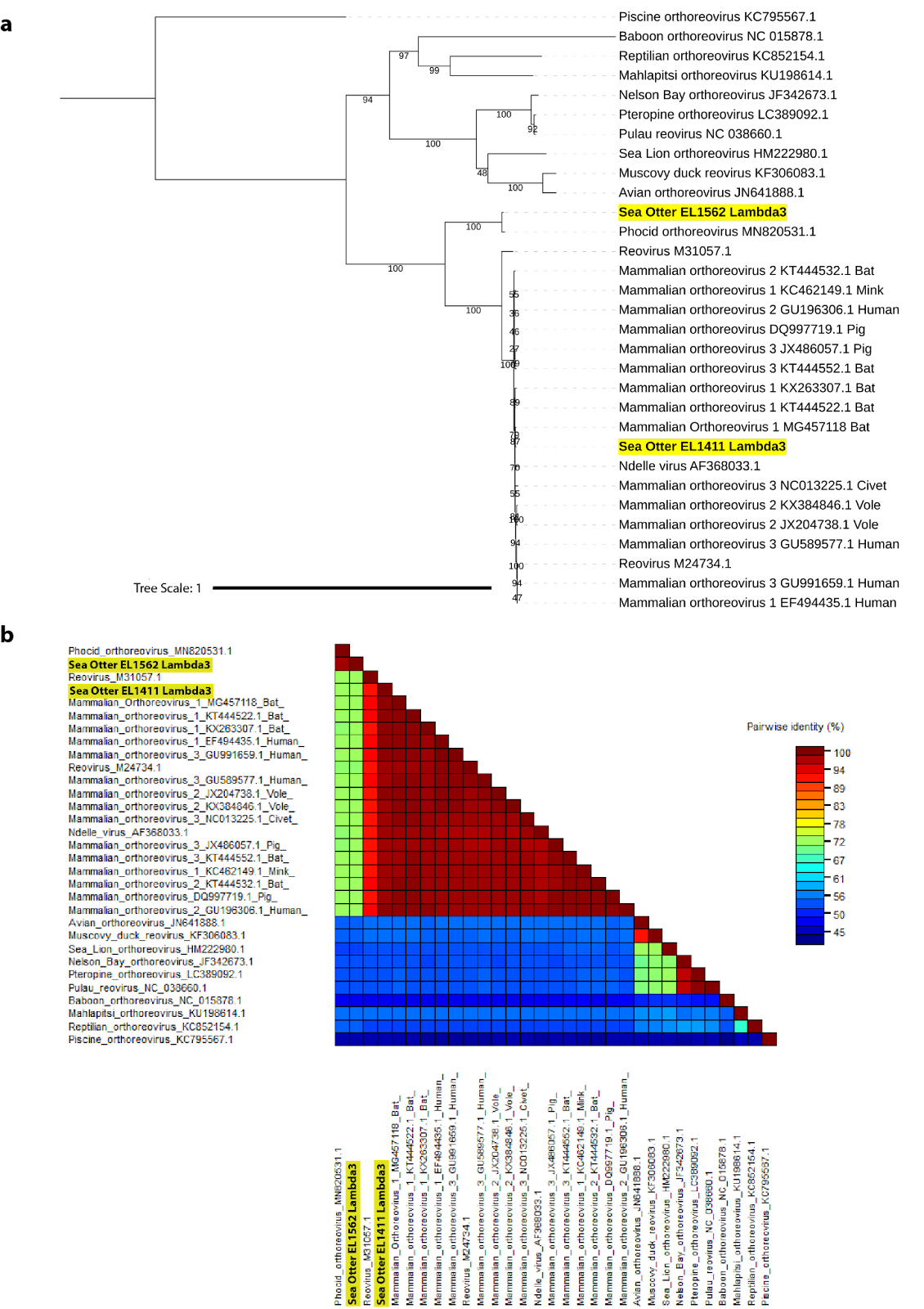
**

Phylogenetic and amino acid identity analysis of sea otter orthoreovirus λ3 amino acid sequences. Maximum likelihood phylogenetic tree of representative strains from representative orthoreovirus species. The tree was constructed based on the amino acid identity of the RNA-dependent RNA polymerase protein for each indicated strain. Novel sequences are indicated in bold with yellow highlights. Phylogenetic analysis was performed with IQTree and visualized with iTOL using Piscine orthoreovirus isolate CGA280-05 as the outgroup. The scale bar represents the estimated average number of aa substitutions per site.

**Fig S4.** µ**1 protein phylogenetic analysis**

**
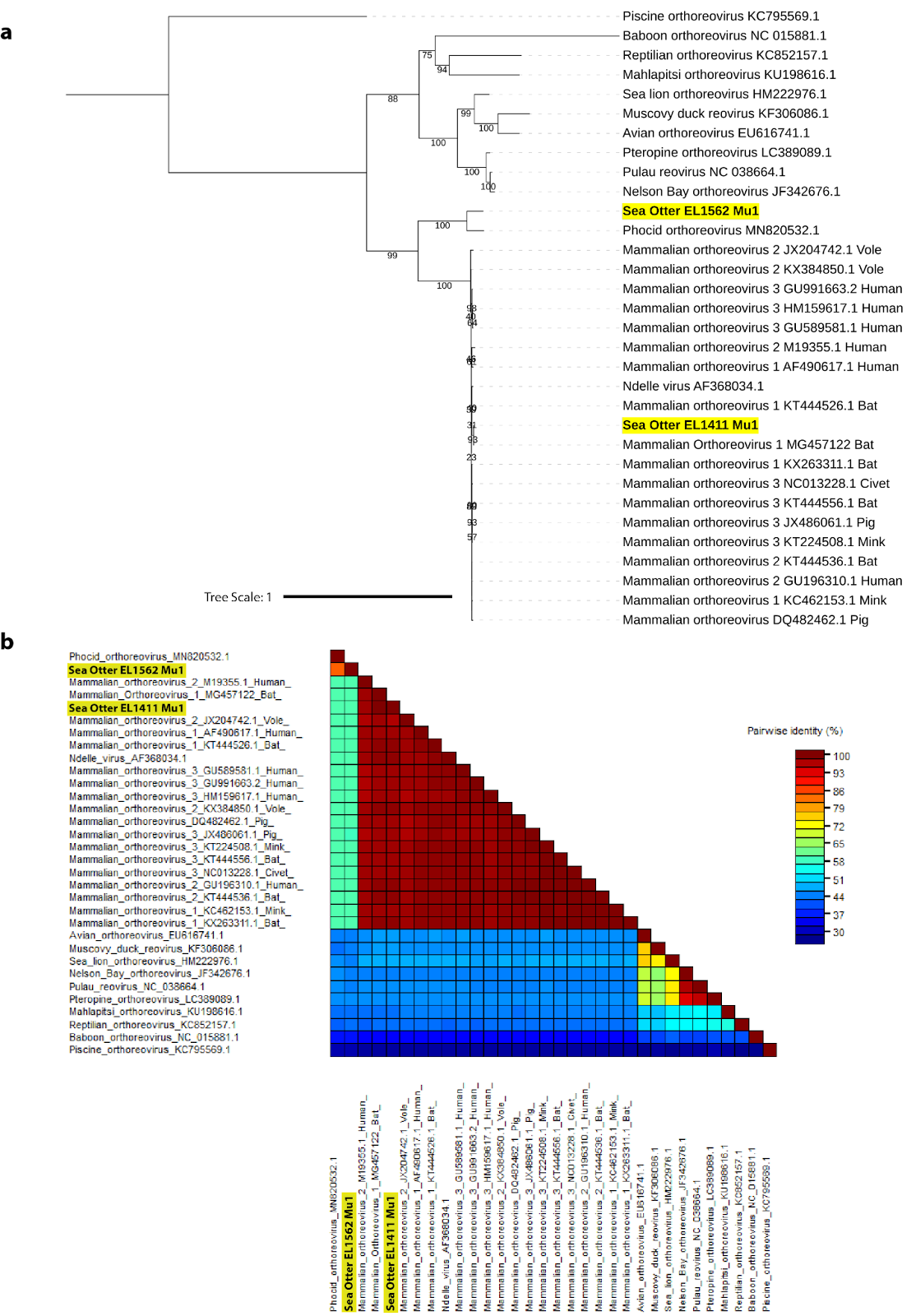
**

Phylogenetic and amino acid identity analysis of sea otter orthoreovirus µ1 amino acid sequences. Maximum likelihood phylogenetic tree of representative strains from representative orthoreovirus species. The tree was constructed based on the amino acid identity of the outer shell protein for each indicated strain. Novel sequences are indicated in bold with yellow highlights. Phylogenetic analysis was performed with IQTree and visualized with iTOL using Piscine orthoreovirus isolate CGA280-05 as the outgroup. The scale bar represents the estimated average number of aa substitutions per site.

**Fig S5.** µ**2 protein phylogenetic analysis**

**
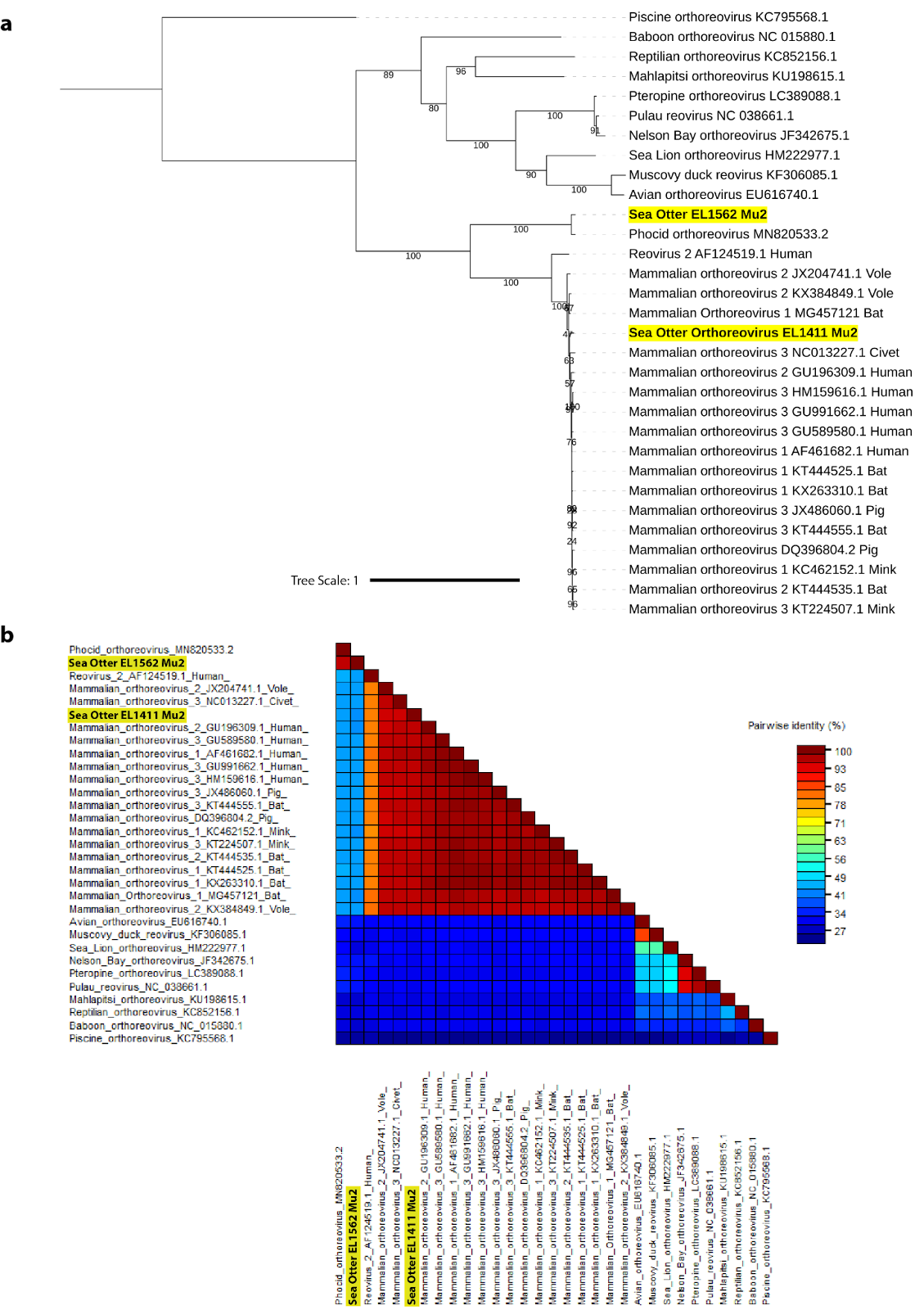
**

Phylogenetic and amino acid identity analysis of sea otter orthoreovirus µ2 amino acid sequences. Maximum likelihood phylogenetic tree of representative strains from representative orthoreovirus species. The tree was constructed based on the amino acid identity of the core NTPase protein for each indicated strain. Novel sequences are indicated in bold with yellow highlights. Phylogenetic analysis was performed with IQTree and visualized with iTOL using Piscine orthoreovirus isolate CGA280-05 as the outgroup. The scale bar represents the estimated average number of aa substitutions per site.

**Fig S6.** µ**NS protein phylogenetic analysis
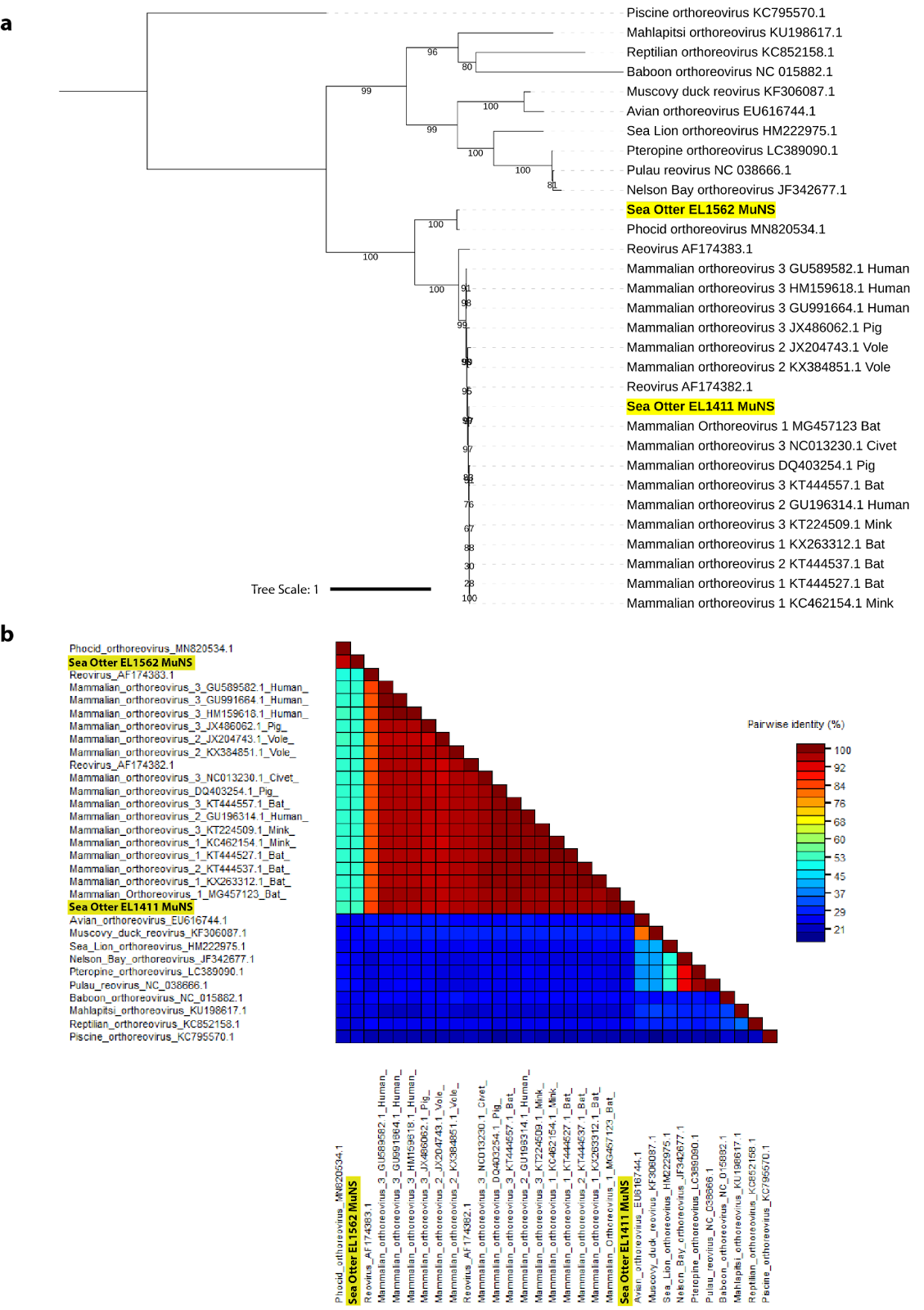
**

Phylogenetic and amino acid identity analysis of sea otter orthoreovirus µNS amino acid sequences. Maximum likelihood phylogenetic tree of representative strains from representative orthoreovirus species. The tree was constructed based on the amino acid identity of the µ non-structural protein for each indicated strain. Novel sequences are indicated in bold with yellow highlights. Phylogenetic analysis was performed with IQTree and visualized with iTOL using Piscine orthoreovirus isolate CGA280-05 as the outgroup. The scale bar represents the estimated average number of aa substitutions per site.

**Fig S7.** σ1 **protein phylogenetic analysis**

**
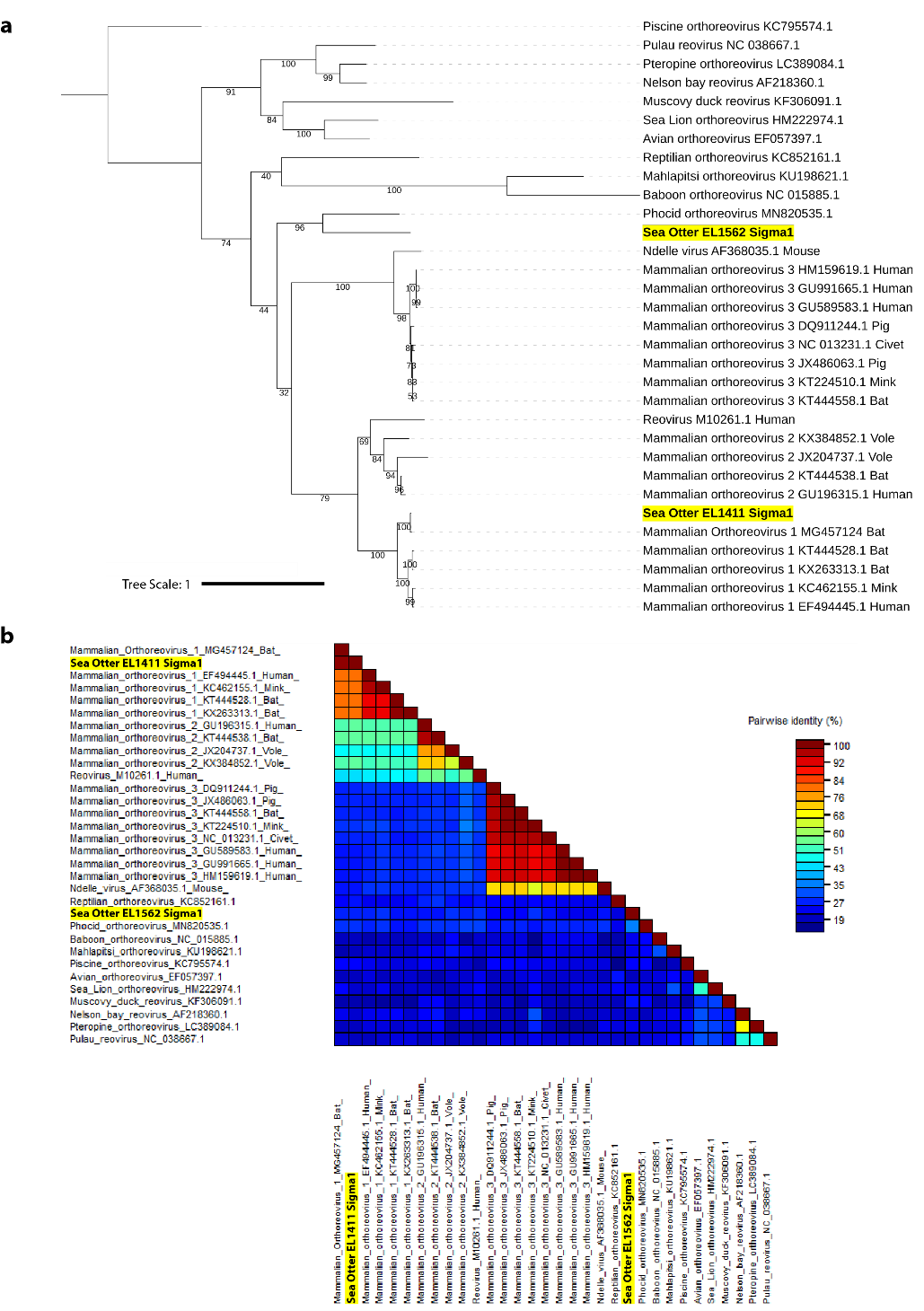
**

Phylogenetic and amino acid identity analysis of sea otter orthoreovirus σ1 amino acid sequences. Maximum likelihood phylogenetic tree of representative strains from representative orthoreovirus species. The tree was constructed based on the amino acid identity of the core clamp protein for each indicated strain. Novel sequences are indicated in bold with yellow highlights. Phylogenetic analysis was performed with IQTree and visualized with iTOL using Piscine orthoreovirus isolate CGA280-05 as the outgroup. The scale bar represents the estimated average number of aa substitutions per site.

**Fig S8.** σ2 **protein phylogenetic analysis**

**
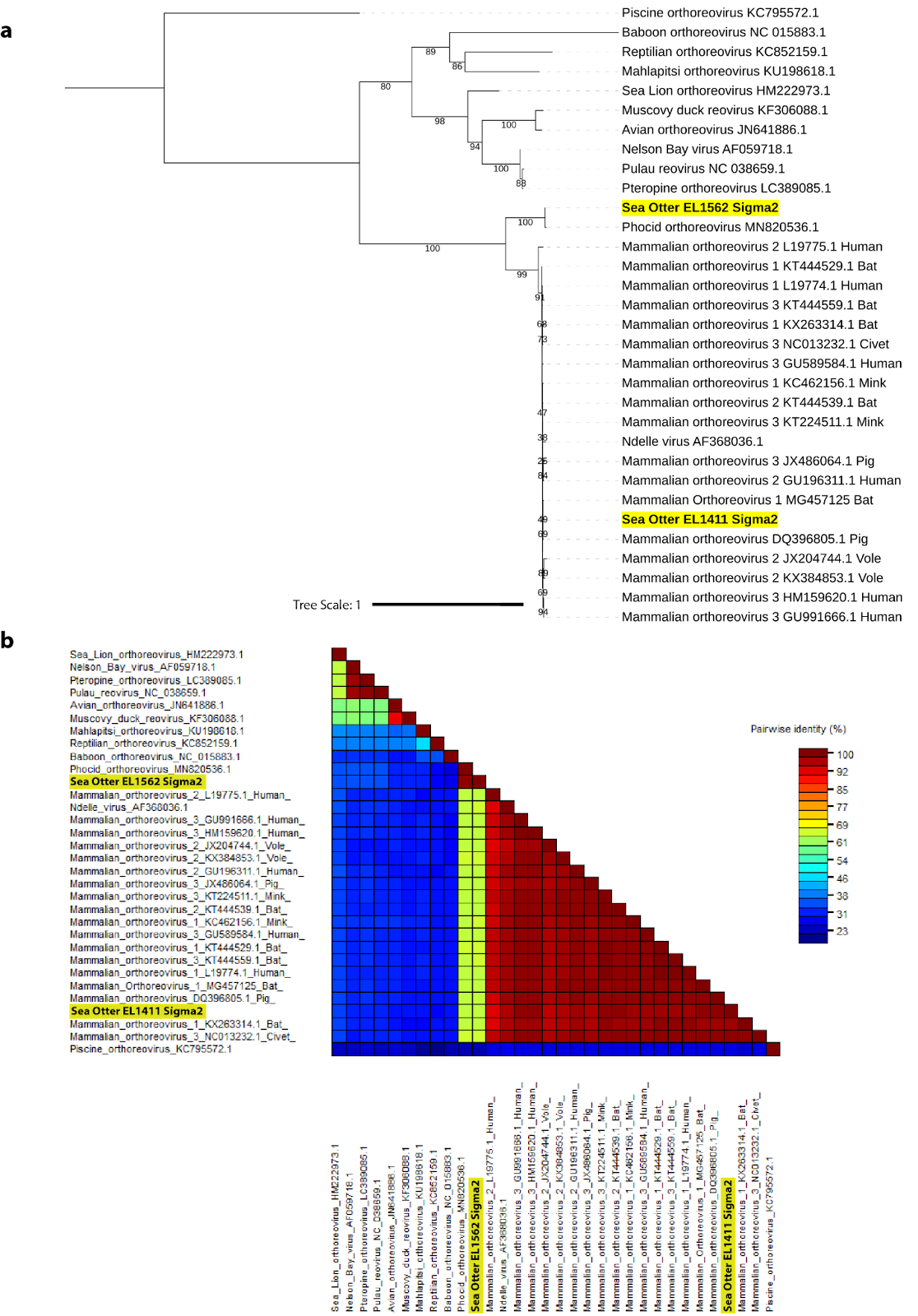
**

Phylogenetic and amino acid identity analysis of sea otter orthoreovirus σ2 amino acid sequences. Maximum likelihood phylogenetic tree of representative strains from representative orthoreovirus species. The tree was constructed based on the amino acid identity of the core clamp protein for each indicated strain. Novel sequences are indicated in bold with yellow highlights. Phylogenetic analysis was performed with IQTree and visualized with iTOL using Piscine orthoreovirus isolate CGA280-05 as the outgroup. The scale bar represents the estimated average number of aa substitutions per site.

**Fig S9.** σ3 **protein phylogenetic analysis**

**
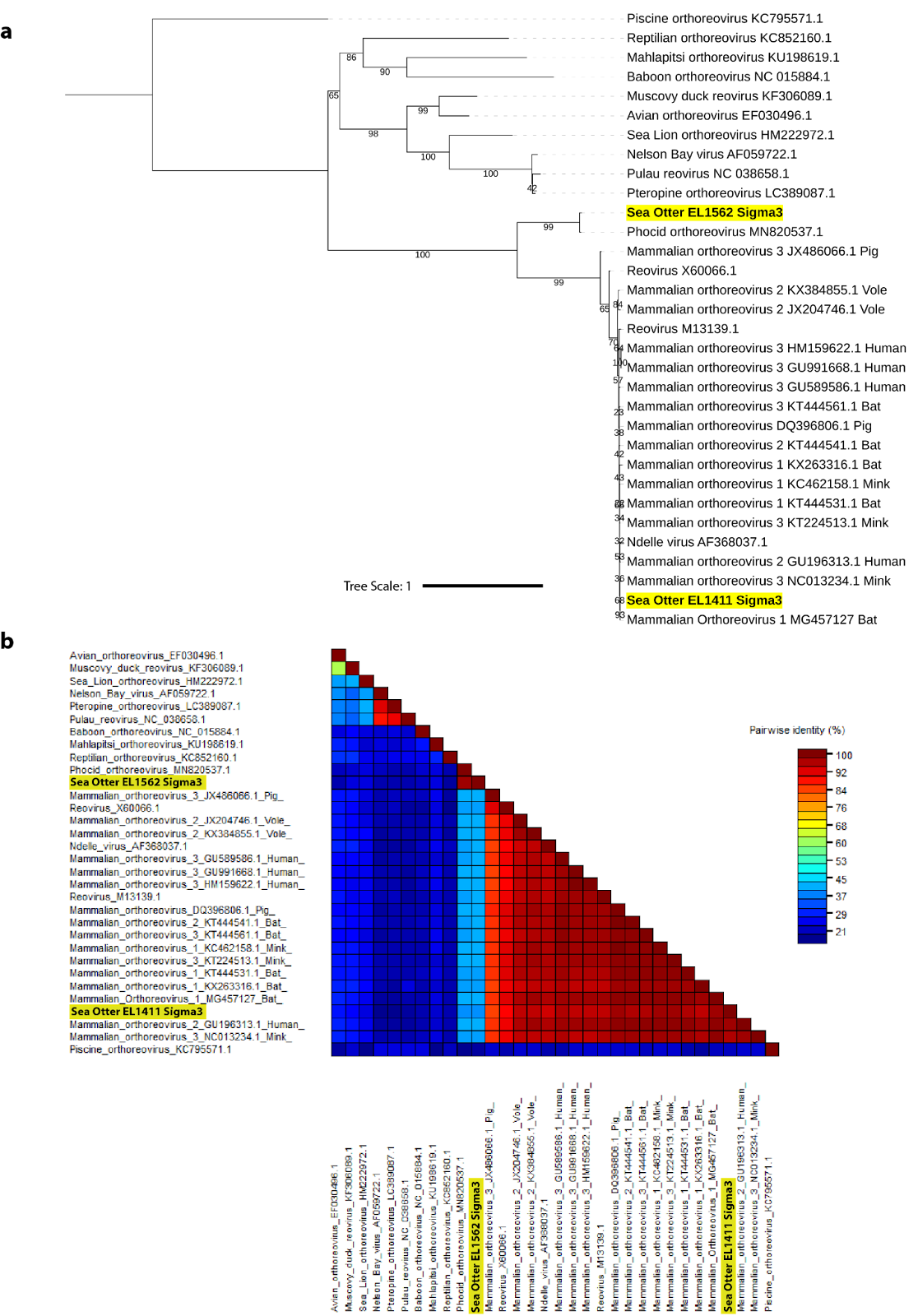
**

Phylogenetic and amino acid identity analysis of sea otter orthoreovirus σ3 amino acid sequences. Maximum likelihood phylogenetic tree of representative strains from representative orthoreovirus species. The tree was constructed based on the amino acid identity of the outer clamp protein for each indicated strain. Novel sequences are indicated in bold with yellow highlights. Phylogenetic analysis was performed with IQTree and visualized with iTOL using Piscine orthoreovirus isolate CGA280-05 as the outgroup. The scale bar represents the estimated average number of aa substitutions per site.

**Fig S10.** σNS **protein phylogenetic analysis**

**
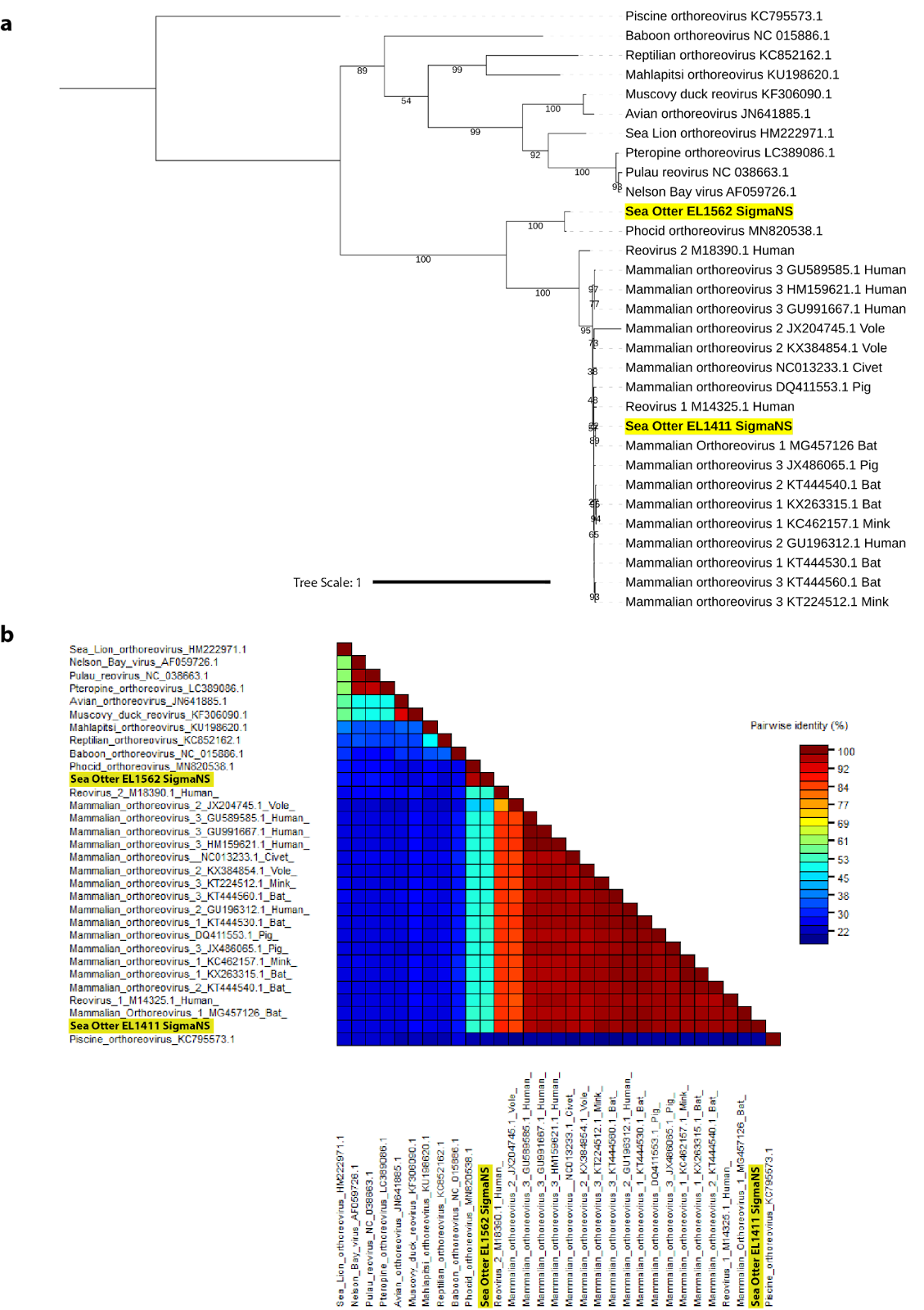
**

Phylogenetic and amino acid identity analysis of sea otter orthoreovirus σNS amino acid sequences. Maximum likelihood phylogenetic tree of representative strains from representative orthoreovirus species. The tree was constructed based on the amino acid identity of the σ non-structural protein for each indicated strain. Novel sequences are indicated in bold with yellow highlights. Phylogenetic analysis was performed with IQTree and visualized with iTOL using Piscine orthoreovirus isolate CGA280-05 as the outgroup. The scale bar represents the estimated average number of aa substitutions per site.
